# Effectiveness of neuromuscular electrostimulation in COPD subjects on mechanical ventilation. A systematic review and meta-analysis

**DOI:** 10.1016/j.clinsp.2022.100108

**Published:** 2022-09-24

**Authors:** Ruvistay Gutiérrez-Arias, Yorschua Jalil, Rocío Fuentes-Aspe, Pamela Seron

**Affiliations:** a Physical Medicine and Rehabilitation Service, Critical Care Unit, Instituto Nacional del Tórax, Santiago, Chile; b Exercise and Rehabilitation Sciences Institute, School of Physical Therapy, Faculty of Rehabilitation Sciences, Universidad Andres Bello, Santiago, Chile; c Departamento Ciencias de la Salud, Carrera de Kinesiología, Facultad de Medicina, Pontificia Universidad Católica de Chile, Santiago, Chile; d Departamento Medicina Intensiva, Facultad de Medicina, Pontificia Universidad Católica de Chile, Santiago, Chile; e Departmento de Ciencias de la Rehabilitación & CIGES, Facultad de Medicina, Universidad de La Frontera, Temuco, Chile

**Keywords:** COPD, Neuromuscular electrostimulation, Mechanical ventilation, Critical illness, Systematic review, Meta-analysis

## Abstract

**Objective:**

To estimate the effectiveness of Neuromuscular Electrostimulation (NMES) in adults with COPD undergoing MV.

**Method:**

A sensitive search was performed in MEDLINE, Embase, CENTRAL, CINAHL and other resources. Randomized Controlled Clinical Trials (RCTs) or non-RCTs that enrolled adults with COPD on MV due to an exacerbation of their disease were included. Two independent reviewers screened, extracted information, and assessed the risk of bias (RoB 2 tool) and the certainty of evidence (GRADE approach) from the included studies.

**Results:**

Four RCTs (144 participants) were included. Subjects who underwent NMES were able to move from bed to chair independently in less time (MD = 4.98 days less; 95% CI -8.55 to -1.47; 2 RCTs; low certainty of the evidence) and they were fewer days on MV (MD = 2.89 days less; 95% CI -4.58 to -1.21); 3 RCTs; low certainty of the evidence) than the control group. However, the effect of NMES on muscle strength is unclear (very low certainty of the evidence).

**Conclusions:**

NMES may improve functional independence and decrease MV time in adults with COPD; however, its effectiveness on muscle strength is uncertain. More and better RCTs are needed to determine with greater certainty the effectiveness of NMES in this population.

## Highlights

• NMES may improve functional independence and decrease in adults with COPD.
• NMES may decrease in duration of MV in adults with COPD.
• More RCTs with a better methodological design are needed.

## Introduction

Chronic Obstructive Pulmonary Disease (COPD), in addition to causing respiratory symptoms derived from restricted airflow [1], is associated with alterations of the peripheral muscles [2,3] possibly resulting from systemic inflammation [4] or physical inactivity [5]. This may be related to a low tolerance to exercise [6], poor functional prognosis, deterioration of quality of life [7], and even an increased risk of mortality [8].

Acute exacerbations of COPD accelerate the progression of the disease, [9] and moderate to severe cases may need to be managed with Mechanical Ventilation (MV). This could complicate the clinical condition of these subjects, with a high rate of failure to wean from MV being reported when it is accompanied by weakness of the appendicular and respiratory muscles [10]. This could produce greater functional deterioration [11], which might extend by several years after hospital discharge [12].

In this context of critical disease, different strategies of early muscle rehabilitation have been proposed [13]. One is Neuromuscular Electrical Stimulation (NMES), an intervention that produces visible contractions on the surface of the muscles [14], thus increasing their oxidative capacity [15] and reducing the inflammatory profile of the subjects [16]. It may also have remote effects on other organs and muscles through the systemic circulation of locally generated myokines 17, 18, 19.

Different systematic reviews have reported increased muscle strength and a shorter stay in the ICU and on MV in critical subjects with a variety of health conditions undergoing NMES 20, 21, 22, 23. In addition, promising effects have been reported specifically in subjects with stable COPD 24, 25, 26. However, the primary studies designed to estimate the effects of NMES on adults with COPD and ventilatory support have not been analyzed consistently, which, added to the contradictory nature of their outcomes, means there is uncertainty as to the effectiveness of NMES in this population. Therefore, the objective of this systematic review is to estimate the effectiveness of NMES in adults with COPD undergoing MV due to an exacerbation of their condition.

## Methods

A systematic review and meta-analysis of controlled clinical trials were conducted. The protocol for this review was registered with the International Platform of Registered Systematic Review and Meta-analysis Protocols (INPLASY) under the number INPLASY202140091, and previously published [27]. The reporting of this review adhered to the statement of Preferred Reporting Items for Systematic Reviews and Meta-Analyses (PRISMA) [28].

## Eligibility criteria

### Types of studies

All controlled clinical trials, whether Randomized (RCTs) or non-RCTs, were included. Studies formally published in scientific journals, peer-reviewed abstracts published in conference proceedings and gray literature were considered. The inclusion of studies was not limited by date or language of publication.

### Type of participants

Studies that enrolled adults (aged 18 years or older) with COPD who were hospitalized and received ventilatory support, either invasive or noninvasive, were included. If the studies enrolled adults with other underlying pathologies other than COPD, they were included only if the data were presented in a disaggregated manner to exclude them from the analysis.

### Type of intervention

Studies that applied Neuromuscular Electrical Stimulation (NMES) were included. This intervention was to be started while COPD subjects were on ventilatory support. Any described modality was considered whether NMES, Transcutaneous Electrical Stimulation (TENS) or Functional Electrical Stimulation (FES), among others. Studies in which the intervention was applied to the muscles of the upper and lower limbs and/or at the thoraco-abdominal level were considered eligible. In addition, the dosage of the sessions (frequency, intensity, duration of each session and of the complete program) did not limit the inclusion of studies in this review.

As for the comparator interventions, studies that did not apply any intervention (passive control) or sham electrical stimulation (placebo control) were considered eligible.

Studies that included other interventions (usual care or standard interventions), such as early mobilization or respiratory muscle training, were excluded from this review if these were not delivered in a manner like the intervention and control groups due to potentially confounding the effect produced by the electrical stimulation.

### Type of outcome

Primary outcomes:

Functional independence, measured by generic or specific validated instruments, for example, the Functional Status Score for the Intensive Care Unit (FSS-ICU) or ICU Mobility Scale (IMS).

Muscle strength, measured through manual assessment, for example, the Medical Research Council Sum-Score (MRC-SS) scale. In addition, the use of dynamometry was also considered, for example, the grip strength.

Duration of MV, measured as the number of days between beginning and end of ventilatory support (invasive or non-invasive MV).

Secondary outcomes:

Duration of MV weaning, measured as the number of days between the beginning of the MV weaning process and the end of ventilatory support (invasive or non-invasive MV).

Dyspnea, measured by any specific validated scale that assesses dyspnea during the performance of activities of daily living, for example, the Medical Research Council dyspnea scale; and during the performance of any functional or maximal exercise test, for example, the Borg scale. In addition, the dyspnea score of any specific quality of life questionnaire for the population with respiratory pathologies was considered, for example, the dyspnea score on the Chronic Respiratory Questionnaire (CRQ).

Fatigue of lower limbs, measured through any specific or validated scale, for example, the Visual Analog Scale (VAS).

Functional exercise capacity, measured by field or functional exercise tests, for example, the 6-minute walk test.

Maximal exercise capacity, measured by laboratory exercise tests, for example, the cardiopulmonary exercise test; incremental shuttle walking test.

Quality of life, measured by generic or specific validated questionnaires, for example, the St. George's Respiratory Questionnaire (SGRQ).

Level of physical activity, measured by means of specific devices, for example, the physical activity monitors or step counters; or by means of specific validated questionnaires, for example, the International Physical Activity Questionnaire (IPAQ).

ICU length of stay, measured as the number of days between admission and discharge from ICU.

Hospital length of stay, measured as the number of days between admission and discharge from the hospital.

Adverse effects, measured through the incidence of any adverse effect directly related to the application of surface muscle electrical stimulation, for example, the allergy in electrode placement, local pain; or other more serious adverse effects, for example, the occurrence of cardiac arrhythmias.

### Information sources and search strategy

The electronic databases consulted up to December 2021 were MEDLINE (through the Pubmed platform), Embase, the Cochrane Library Clinical Trials Register (CENTRAL) (through the Cochrane Library platform), and Cumulative Index to Nursing and Allied Health Literature (CINAHL) (through the EBSCOhost platform). The keywords used consisted of MESH and EMTREE terms according to the database used, in combination with free keywords using Boolean “AND” and “OR” operators (see the supplementary materials).

In addition, two clinical trial registries were reviewed (International Clinical Trials Registry Platform (ICTRP) and ClinicalTrials.gov of the U.S. National Library of Medicine), and a gray literature publication platform (http://opengrey.eu/search/) was queried (see the supplementary materials).

Reference lists of included studies and previously published systematic reviews [20, 24, 25, 26] were also hand searched. No language, publication status, or date restrictions were applied.

### Study selection and data extraction

Two reviewers (RGA and YJ) independently assessed that each record identified by the search met the eligibility criteria. The Rayyan® app was used for this stage [29]. For data extraction, two independent reviewers (RGA and YJ) used a standard form to obtain the general information and characteristics of each included study. Disagreements were resolved by consensus or ultimately by a third reviewer (RF or PS).

### Risk of bias and certainty evidence assessment

Two reviewers (RGA and YJ) assessed the risk of bias in the estimate of each outcome reported by the included studies using the second version of the Cochrane risk of bias tool (RoB 2). According to this tool, studies were categorized as “low risk”, “some concerns” or “high risk” of bias. In addition, the certainty of the evidence of the estimate of each outcome reported was assessed independently by two reviewers (RGA and PS) using the GRADE approach [30], which was presented in a “Summary the Findings (SoF)” Table [31,32]. Disagreements were resolved by consensus.

### Data synthesis and analysis

The characteristics of the included studies were described qualitatively. The estimation of the intervention effect was performed quantitatively by a meta-analysis with a random effects model. Since all the outcomes were continuous, Mean Differences (MD) were calculated with their respective 95% CI. When the results were reported as medians with p25-p75 or minimum-maximum, these statistics were used to estimate the mean and Standard Deviation (SD) [33].

Heterogeneity was analyzed using the Chi^2^ test with N-1 degrees of freedom, with an alpha of 0.05 as the threshold for statistical significance, and the I^2^ test. A value between 25% and 50%, 50% and 75%, and greater than 75% of I^2^ corresponds to low, moderate, and high levels of heterogeneity respectively [34]. The Review Manager software (Version 5.4.1; Cochrane, Oxford, United Kingdom) was used.

Publication bias was assessed by visualizing a funnel plot, and Begg's and Egger's tests for the possible existence of small study bias using the RStudio software (Version 2021.09.0©, 2009‒2021 RStudio, PBC).

## Results

### Search results

**Fig. 1:**
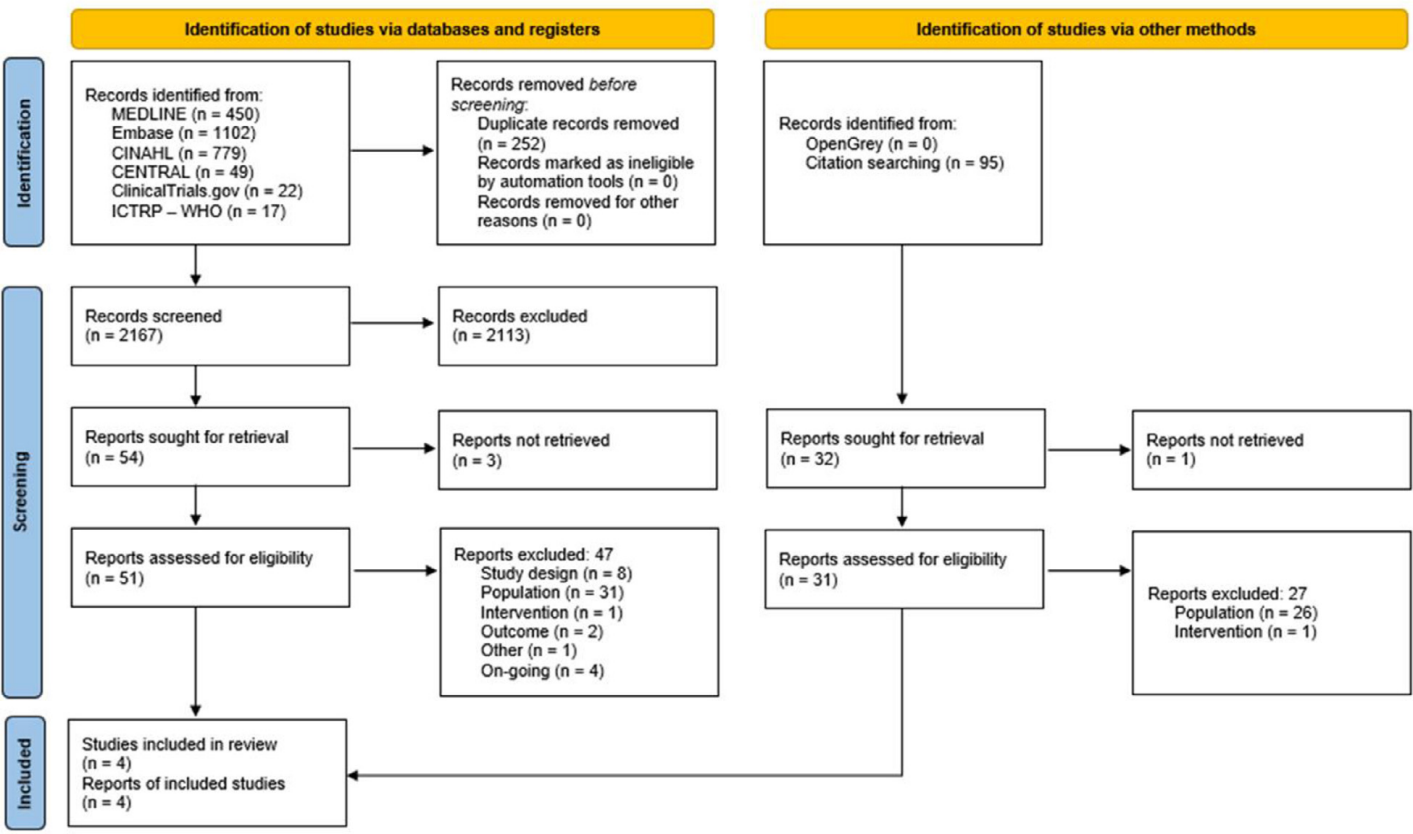
Study selection flowchart.

A total of 2419 records were identified through the electronic search. After removing duplicates, 2167 records were screened, of which 51 were evaluated in full text. Of these, 47 records were excluded mostly because of the type of population (subjects with COPD not undergoing MV), and in addition, 4 are ongoing studies that could be considered for future updates of this review (see the supplementary materials). Finally, 4 records, corresponding to 4 different studies 35, 36, 37, 38, met the eligibility criteria of this review (Fig. 1).

In addition, 95 unique records were identified through other search resources, of which 31 were evaluated in full text, identifying the same 4 included records/studies from the databases and registers search (Fig. 1).

Records not retrieved in full text and the reasons for exclusion of records assessed in full text are presented in supplementary materials.

### Characteristics of the included studies

The four studies included in this review had an RCT design. Three of them included two groups [35,37,38] and one included 3 parallel groups [36]. Two of them were conducted in Turkey [36,37], one in Italy [35] and one in China [38].

In total, the studies included 144 participants, of which 134 were analyzed in this review because only 2 of the 3 groups included in the Akar 2015 study [36] were considered in order to isolate the effect of the NMES. The mean age of the participants was 65.1 years (95% CI 62.6 to 67.6), and 63.4% were male. In all studies subjects underwent invasive MV, three via orotracheal tube 36, 37, 38, and one via tracheostomy [35].

Three studies were conducted in an acute critical care setting 36, 37, 38, while one study was conducted in a chronic critical care rehabilitation setting in which participants with COPD with more than 30 days in bed were referred to a specialized center to continue their rehabilitation process [35].

Details of the participant characteristics and the interventions delivered to the intervention and control groups are shown in Table 1.

**Table 1:** Characteristics of the included studies.

ID	Groups		Interventions	
	Experimental	Control	Experimental	Control
Zanotti 2003	*n* = 12	*n* = 12	Stimulated muscles: quadriceps and vastus glutei	Active mobilization of 4 limbs as soon as possible
	Age, y: 66.2 ± 8	Age, y: 64.5 ± 4		
	Male, n (%): 8 (66.7%)	Male, n (%): 9 (75%)		
	BMI, kg/m^2^: 24.5 ± 2.4	BMI, kg/m^2^: 22.4 ± 3.7	Device: SportTrainer; Actionfit; Forlì, Italy	
	Barthel, p: NR	Barthel, p: NR		
	APACHE II, p: NR	APACHE II, p: NR	Dosage: 5 m at 8 Hz pulse width 250 ms and then 25 m at 35 Hz pulse width 350 ms	
			Sessions: maximum time tolerated by the patient and gradually increasing the time of training up to 30 m; 5 d/w; 4w	
			* In addition, the same interventions that were delivered to the control group were also applied	
Akar 2015	*n* = 10	*n* = 10	Stimulated muscles: Deltoids and quadriceps	Active exercises of 4 limbs. If active exercise was not possible, patients underwent passive or assisted active exercise
	Age, y: 70 ± 12.3	Age, y: 68 ± 17.8		
	Male, n (%): 4 (40%)	Male, n (%): 5 (50%)	Device: COMPEX (MI theta PRO, Switzerland)	
	BMI, kg/m^2^: NR	BMI, kg/m^2^: NR		
	Barthel, p: NR	Barthel, p: NR	Dosage: 20‒25 mA; 50 Hz; 6 s of contraction duration, 1.5 s of increase and 0.75 s of decrease	
	APACHE II, p: NR	APACHE II, p: NR		
			Sessions: 5 d/w; 20 sessions	
			* In addition, the same interventions that were delivered to the control group were also applied	
Koçan 2015	*n* = 15	*n* = 15	Stimulated muscles: pectorals major, trapezius and latissimus dorsi	Upper extremity range-of-motion exercises and controlled breathing techniques and respiratory physiotherapy
	Age, y: 66.1 ± 13.9	Age, y: 69.9 ± 11		
	Male, n (%): 14 (93.3%)	Male, n (%): 14 (93.3%)		
	BMI, kg/m^2^: 26.6 ± 7.4	BMI, kg/m^2^: 25.7 ± 4.5	Device: COMPEX (MI theta PRO, Switzerland)	
	Barthel, p: NR	Barthel, p: NR		
	APACHE II, p: NR	APACHE II, p: NR	Dosage: 20‒25 mA; 50 Hz; 6 s of contraction duration, 1.5 s of increase and 0.75 s of decrease	
			Sessions: 20 m/d; 1d	
			* In addition, the same interventions that were delivered to the control group were also applied	
	*n* = 30	*n* = 30	Stimulated muscles: Biceps, triceps, quadriceps, tibialis anterior and gastrocnemius	Usual treatment that includes etiological treatment and general symptomatic support, in addition to passive and active functional training and pulmonary rehabilitation
	Age, y: 62.4 ± 13.6	Age, y: 59.8 ± 11.8		
	Male, n (%): 14 (51.9%)	Male, n (%): 17 (58.6%)		
			Device: KT90A (Beijing Yaoyang Kangda Medical Co)	
	BMI, kg/m^2^: 22.3 ± 1.5	BMI, kg/m^2^: 22.5 ± 1.6		
	Barthel, p: 89.4 ± 6	Barthel, p: 90.1 ± 5.3		
	APACHE II, p: 19.8 ± 4.4	APACHE II, p: 19.0 ± 4.2	Dosage: 30‒40 Hz	
			Sessions: 30 m; 2/d; until the patient is transferred from ICU	
			* In addition, the same interventions that were delivered to the control group were also applied	

Note: 1) Akar 2015 included 3 groups, of which two (Group 1: exercise training + electrostimulation; Group 3: exercise training) were considered in the analysis performed in this review; 2) Chen 2019 measured Barthel index two weeks before ICU admission.

APACHE II, Acute Physiology and Chronic Health disease Classification System II; BMI, Body Mass Index; d, days; Hz, Hertz; ICU, Intensive Care Unit; m, minutes; mA, milliamps; ms, microseconds; NR, Not Reported; p, points; s, seconds; w, weeks; y, years.

### Risk of bias assessment

**Fig. 2:**
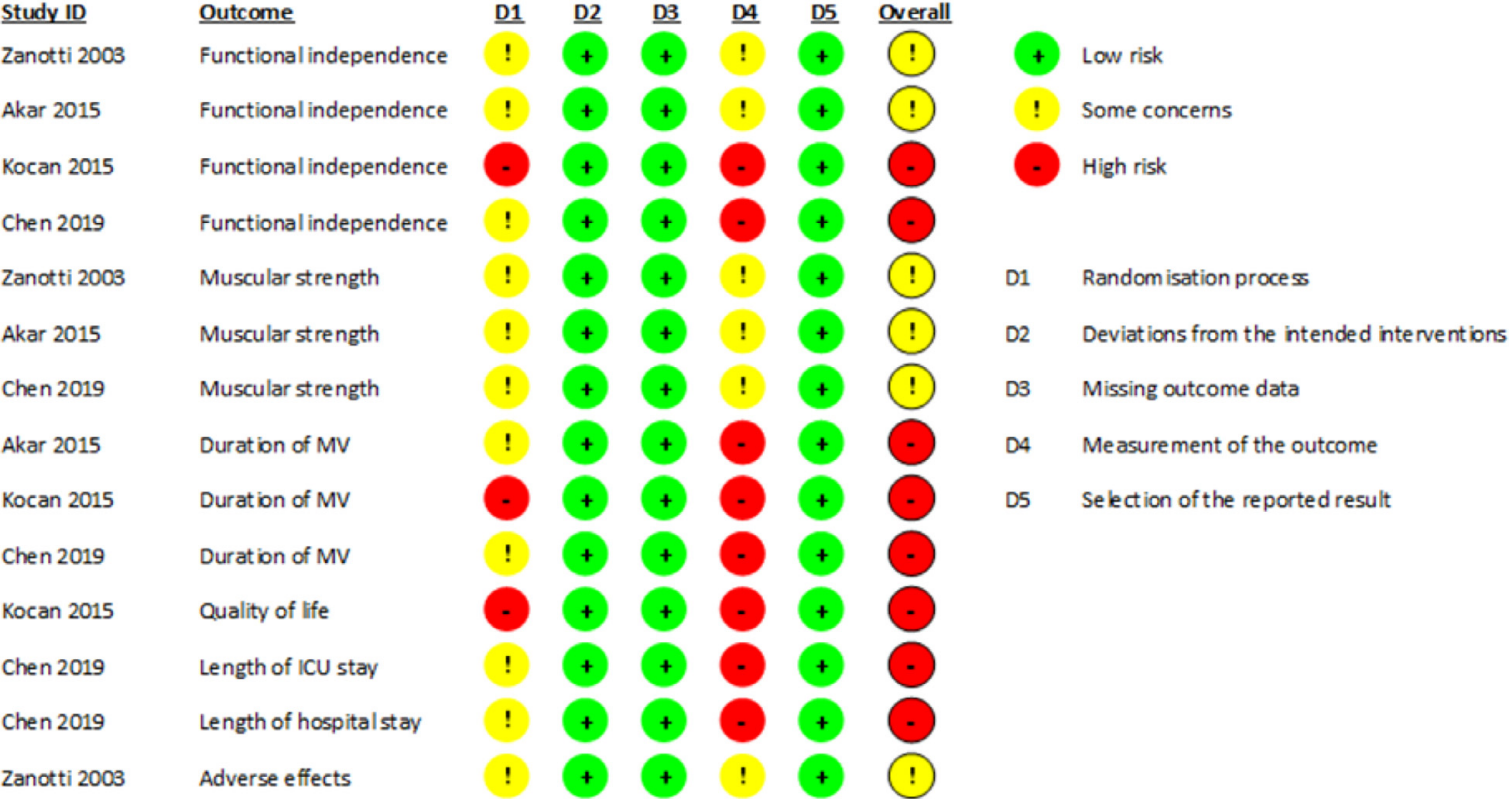
Risk of bias of reported outcomes. ICU, Intensive Care Unit; MV, Mechanical Ventilation.

The overall risk of bias in the estimate of the effect of NMES on the 7 outcomes reported by the included studies was rated as “some concerns” or “high” (Fig. 2).

The rating of “some concerns” was because the studies did not specify the method of randomization or whether this process was concealed, and because the outcome assessors were not blinded, associated with the unlikelihood that this lack of blinding could influence the assessment of outcomes.

The rating of “high” was either because the randomization process described by one of the studies was alternation and therefore not concealed [37], or because the outcome assessors were not blinded, which is associated with the high likelihood that this lack of blinding may influence the outcome assessment because the outcome assessment was performed by the study participants themselves or because the outcome assessor is the care provider making the decision.

### Effects of interventions and certainty of the evidence

The studies included in this review reported outcome data for the 3 primary outcomes (functional independence, muscle strength, and duration of MV), and for quality of life, length of ICU and hospital stay, and adverse effects. The certainty of the evidence for these 7 outcomes is shown in a SoF table (Table 2).

**Table 2:** Summary of findings.

Outcomes	Illustrative comparative risks*		N° of participants (studies)	Certainty	Comments
	Assumed risk Control	Corresponding risk NMES (95% CI)			
Functional independence (Time to move from bed to chair) ‒ days	The mean range of time across the control groups was from 12.6 to 14.33	MD 4.86 lower (8.55 to 1.47 lower)	44 (2 studies)	⊕⊕⊝⊝ Low^a^	A third study reported no difference in the FIM scale between the groups, and the fourth study reported a higher Barthel index score in the intervention group
Muscular strength (manual evaluation) ‒ points	1 study reported no difference between the groups and 2 studies reported greater muscle strength in the intervention group		104 (3 studies)	⊕⊝⊝⊝ Very low^b^	One study also reported greater grip strength in the intervention group.
					Due to the way in which the results were reported it was not possible to perform a meta-analysis
Duration of MV ‒ days	The mean range of time across the control groups was from 6.75 to 18.13	MD 2.89 lower (4.58 to 1.21 lower)	96 (3 studies)	⊕⊕⊝⊝ Low^a^	One study reported that one-third of all patients were unable to wean from MV
Quality of life (SGRQ and SF-36) ‒ points	1 study reported no difference between the groups		30 (1 study)	⊕⊕⊝⊝ Low^a^	The study does not report the scores of the questionnaires, and the graphs of the SGRQ tool do not allow to extract the information
Length of ICU stay ‒ days	The mean range of time across the control groups was from 7.5 to 10.45	MD 4.2 higher (9.1 lower to 17.5 higher)	69 (2 studies)	⊕⊝⊝⊝ Very low^b^	One study reported that one third of all patients were unable to be discharged from ICU
Length of hospital stay ‒ days	The main of the control group was 15.38	MD 2.17 lower (3.74 to 0.6 lower)	60 (1 study)	⊕⊕⊝⊝ Low^a^	
Adverse effects	1 study reported that the interventions were well tolerated by both groups		24 (1 study)	⊕⊕⊝⊝ Low^a^	The remaining 3 studies make no mention of the safety or tolerability of NMES and the control intervention.

⁎ The corresponding risk (and its 95% CI) is based on the assumed risk in the comparison group.

a The certainty of the evidence was downgraded by the risk of bias of the included studies and by imprecision (sample size less than the optimal information size).

b The certainty of the evidence was downgraded by the risk of bias of the included studies, by imprecision (sample size less than the optimal information size), and by inconsistency. 95%CI, Confidence Interval 95%; FIM, Functional Independence Measure; MD, Mean Difference; MV, Mechanical Ventilation; NMES, Neuromuscular Electrostimulation; SGRQ, Saint George's Respiratory Questionnaire; SF-36, Short Form 36 Health Survey Questionnaire. GRADE Working Group grades of evidence: ⊕⊕⊕⊕ High certainty: We are very confident that the true effect lies close to that of the estimate of the effect. ⊕⊕⊕⊝ Moderate certainty: We are moderately confident in the effect estimate: The true effect is likely to be close to the estimate of the effect, but there is a possibility that it is substantially different. ⊕⊕⊝⊝ Low certainty: Our confidence in the effect estimate is limited: The true effect may be substantially different from the estimate of the effect. ⊕⊝⊝⊝ Very low certainty: We have very little confidence in the effect estimate: The true effect is likely to be substantially different from the estimate of effect.

None of the studies included in this review reported results on the duration of MV weaning, dyspnea, fatigue of lower limbs, functional exercise capacity at discharge, maximal exercise capacity at discharge, or physical activity level at discharge.

### Primary outcomes

#### Functional independence

The 4 studies included in the review reported this outcome using different forms of assessment 35, 36, 37, 38. Two studies reported the time to reach different motor milestones [35,36], one study reported the Functional Independence Measure (FIM) [37], and one study reported the Barthel Index [38].

**Fig. 3:**
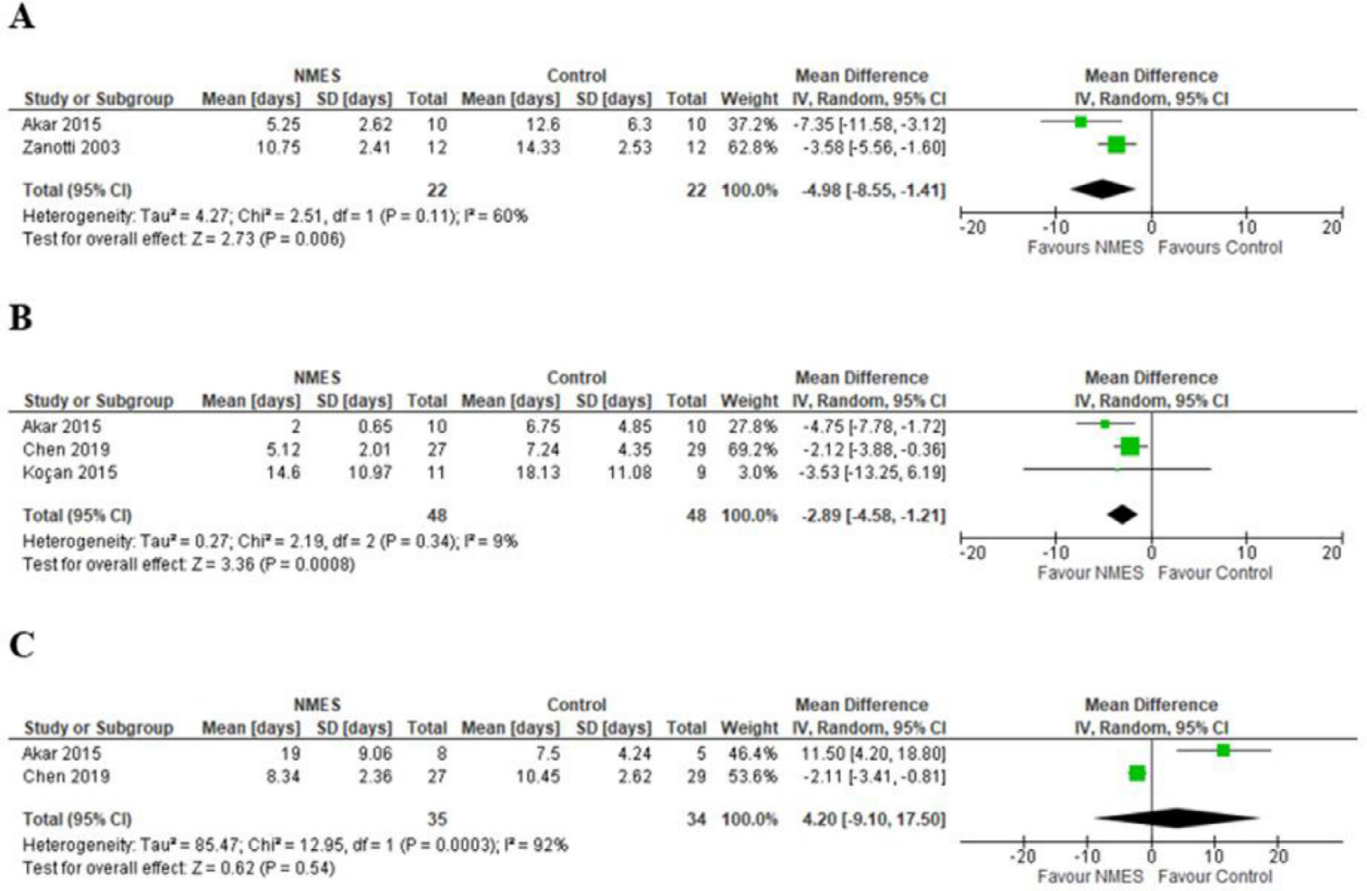
Pooled analysis. (A) Functional independence (Time to move from bed to chair); (B) Duration of MV; (C) Length of stay in ICU.

Pooled analysis of two studies (44 participants) [35,36] showed that the group undergoing NMES achieved transfer from bed to chair independently in 4.98 days less (95% CI −8.55 to −1.47) than the control group (low certainty of evidence; Table 2). The level of heterogeneity of the studies was moderate (I^2^ = 60%; *p* = 0.11) (Fig. 3A). In addition, one study (20 participants) [36] reported that there was no difference between the groups in time to achieve other motor milestones (*p* > 0.05).

Additionally, one study (30 participants) reported that both the NMES group and the control group had significantly improved FIM scores, without mentioning the comparison between the groups (no p-values reported) [37], and another study (60 participants) reported that the experimental group had a Barthel Index score at ICU discharge of 3.91 points higher (95% CI 1.52 to 6.30) than the control group [38].

#### Muscle strength

Three studies reported this outcome [35,36,38], which measured muscle strength manually using the MRC-SS scale or similar, and in addition, one of them measured grip strength by dynamometry [38].

One study (20 participants) reported that the experimental group significantly increased muscle strength in the upper (*p* = 0.04) and lower limbs (*p* = 0.01), while the control group only experienced this increase in the upper limbs (*p* = 0.03). However, there were no differences between the groups (p-value not reported) [36]. Another study (24 participants) reported that the NMES group increased their muscle strength 0.91 points more (95% CI 0.19 to 1.63) than the control group [35]. Finally, the third study (60 participants) reported that the experimental group showed 9.65 points more (95% CI 5.49 to 13.81) on the MRC-SS scale, and 6.04 kg more (95% CI 0.43 to 11.65) on the grip strength than the control group [38] (very low certainty of evidence; Table 2).

Due to the way in which the results were reported (MRC-SS, mean with a standard deviation of all measurements, and median with min-max separated by upper and lower limbs), it was not possible to perform a meta-analysis.

#### Duration of MV

Three studies reported this outcome 36, 37, 38, of which one reported that at the end of the study (30 days duration) 6 participants in the control group and 4 in the experimental group were unable to wean from MV [37].

Pooled analysis of the three studies (96 participants) showed that the group undergoing NMES had a shorter MV duration, with 2.89 days less (95% CI −4.58 to −1.21) than the control group (low certainty of evidence; Table 2). There was no heterogeneity among the studies (I^2^ = 9%; *p* = 0.34) (Fig. 3B).

One study only reported that at the end of the study, 18 of the 24 participants were weaned from MV without specifying the group to which they belonged [35].

### Secondary outcomes

#### Quality of life

Only one study (30 participants) reported on the quality of life, which was measured using the Saint George's Respiratory Questionnaire (SGRQ) and Short Form 36 Health Survey Questionnaire (SF-36) tools [37].

All the parameters on the SGRQ were not significantly different at day 30 between the two groups (*p* > 0.05). Regarding the SF-36 questionnaire, the scores of physical function, general health, vitality, social function, and mental health improved significantly in both groups (*p* < 0.05), while body pain only improved in the intervention group (*p* = 0.03), and emotional role only improved in the control group (*p* = 0.02) [37] (low certainty of evidence; Table 2). However, no differences were reported between the groups at the end of the study (p-value not reported).

#### Length of ICU stay

Two studies reported this outcome [36,38], of which one reported that at the end of the study 2 participants in the experimental group and 5 in the control group were not discharged from the ICU [36].

Pooled analysis of the two studies (69 participants) showed no difference in ICU length of stay between the groups (mean difference = 4.2 days (95% CI −9.1 to 17.5)) (very low certainty of evidence; Table 2). The level of heterogeneity of the studies was high (I^2^ = 92%; *p* < 0.001) (Fig. 3C).

#### Length of hospital stay

Only one study (60 participants) reported the length of hospital stay [38]. The hospital length of stay of the experimental group was on average 2.17 days less (95% CI −3.74 to −0.60) than the control group (low certainty of evidence; Table 2).

#### Adverse effects

Only one study (24 participants) mentioned that both the control and experimental groups tolerated the interventions adequately and reported that no deaths occurred during the study [35]. In addition, only one study (60 participants) presented dropouts of participants during the study due to economic reasons (control group: 1; experimental group: 2), and serious illness (experimental group: 1) which was not attributed to the application of NMES [38] (low certainty of evidence; Table 2).

### Publication bias

**Fig. 4:**
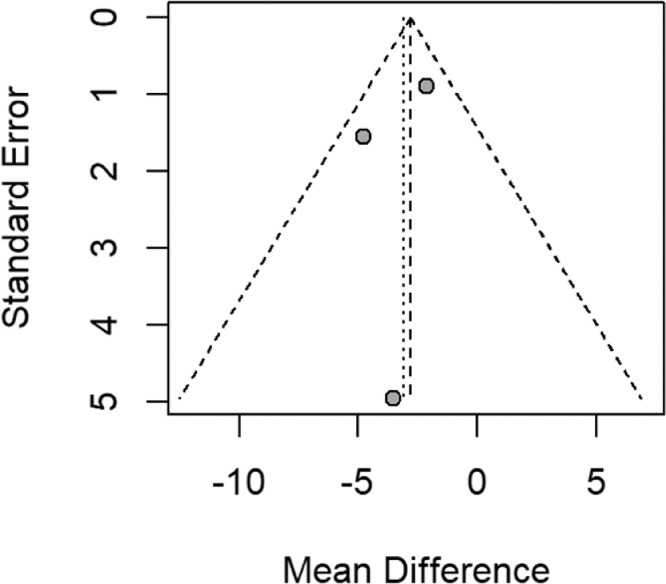
Funnel plot – duration of MV.

The funnel plot (Fig. 4), Begg's test (*p* = 0.60), and Egger's test (*p* = 0.66) indicate that there was no reporting bias in the estimation of the duration of MV.

Given that the estimation of the effect of NMES on functional independence and length of ICU stay only incorporated two studies in the meta-analysis, and that the rest of the outcomes could not be pooled, the possible small study effect could not be determined.

## Discussion

Four RCTs fulfilled the eligibility criteria of this review 35, 36, 37, 38, including 134 participants with COPD subjected to invasive MV who underwent NMES or some control intervention, either habitual care or simulated NMES.

The results of this review show that NMES can improve functional independence expressed in less time than subjects with COPD moved independently from bed to chair. This could be due to NMES increasing muscle strength in critical subjects [23] and in subjects with COPD not on MV [24], and to the positive relation between muscle strength and functionality [39,40]. However, according to this review, the effects of NMES on muscle strength are unclear, because there is no single estimator of effectiveness through a meta-analysis as a result of the inadequate reporting of outcome data provided by two of the included studies [35,36], considering that the study by Chen et al. reported a significantly higher score on the MRC-SS scale in the experimental group compared to the control [38], which has also been reported in subjects on prolonged mechanical ventilation with or without COPD [41]. In any case, for this last outcome it must be considered that there is an altered response of muscle fibers in subjects with COPD to a contractile stimulus [42], which could cause differing degrees or times for recovery compared to critical subjects without COPD as the underlying disease.

In addition, the results of this review showed that NMES can reduce exposure time to MV, a relevant finding in the context of the complications that could occur on prolonged MV [43]. Although in the studies included in this review NMES was applied in greater proportion to the peripheral muscles [35,36,38] and less to the accessory respiratory muscles [37], the effect of reducing the time on MV could be due to the remote effect that NMES might have through the release of myokines into the systemic circulation [44] given their potential effect on other muscles not subjected to NMES, such as the diaphragm.

Yet despite only one study included in this review reported that subjects with COPD undergoing NMES tolerated it adequately [35], these findings are similar to those in the critical population in general [45]. Thus, this intervention could be safe for this population as long as the contraindications for their use are considered [46], aspects that were considered by all the studies at the time of recruiting their participants 35, 36, 37, 38.

Despite the possible effects on functional independence and MV time estimated by this review, NMES may have no effect on the quality of life, a finding reported previously by systematic reviews that included subjects with COPD not necessarily placed on MV [24,25]. In addition, it may be possible to expect subjects with greater functionality and lower exposure time to MV to stay less time in the ICU and the hospital; [47] however, due to the low-very low certainty of the evidence that these outcomes report, this cannot be confirmed by the findings of this review. Future studies that intend to report these outcomes would have to consider the point at which subjects are in a condition to be discharged from the ICU or the hospital, and not when they are actually discharged, since this measurement could be biased by administrative or organizational topics depending on the country or context in which they are carried out [48].

The risk of bias in the studies included in this review was described as “some concerns” or “high” for all the reported outcomes. Together with the few participants included in the study, this contributed to the low certainty of the evidence for functional independence, length of stay on MV, quality of life, length of hospital stays, and incidence of adverse effects. This indicates that future studies will very likely have a significant impact on the authors’ confidence in the estimation of the effect, and it is probable that this estimation will change [30]. On the other hand, if the inconsistency in some reported results and the very low certainty of the evidence for muscle strength and duration of ICU stay is considered, any estimation of the effect is very uncertain [30].

### Limitations of the review

It must be noted that two of the studies included in this review reported the results for length of time on MV [36] and length of hospital stay [37] using median and minimum-maximum so the mean and SD had to be estimated from these data, which can have a small margin of error [33].

In addition, it must be considered that applying the methods used to determine the presence of publication bias (funnel plot and statistical tests), it is recommended that at least 10 studies be included so their valuation is more reliable [49], bearing in mind that a maximum of three studies were available for this estimation in the length of time on MV.

## Conclusion

NMES may improve functional independence, decrease in duration of MV, and be safe in adults with COPD. However, it may have no effect on the quality of life or hospital length of stay, and its effectiveness on muscle strength and ICU length of stay is uncertain. Duration of MV weaning, dyspnea, fatigue of lower limbs, functional exercise capacity at discharge, maximal exercise capacity at discharge, and physical activity level at discharge was not measured in the included studies.

More RCTs with a better methodological design are needed to estimate with greater certainty the real effectiveness of NMES in this population so that the use of this intervention can be recommended with greater confidence.

## Authors' contributions

Literature search: Ruvistay Gutiérrez-Arias.

Data collection: Ruvistay Gutiérrez-Arias and Yorschua Jalil.

Study design: Ruvistay Gutiérrez-Arias, Yorschua Jalil, Rocío Fuentes-Aspe and Pamela Seron.

Analysis of data: Ruvistay Gutiérrez-Arias and Yorschua Jalil.

Manuscript preparation: Ruvistay Gutiérrez-Arias.

Review of manuscript: Yorschua Jalil, Rocío Fuentes-Aspe and Pamela Seron.

## Funding

No external funding.

## Conflicts of interest

The authors declare no conflicts of interest.

## Appendix Supplementary materials

Image, application 1
