# Supplementary material for: Effectiveness of neuromuscular electrostimulation in COPD subjects on mechanical ventilation. A systematic review and meta-analysis
Source: Clinics (Sao Paulo). 2022 Sep 24;77:100108. doi: 10.1016/j.clinsp.2022.100108 (PMC9513212; doi:10.1016/j.clinsp.2022.100108)
Supplement: Supplementary file 1 [file mmc1.pdf]

Online resource 1. Search strategy

Electronic databases

Database		Search Term
MEDLINE (Pubmed)	1	"Pulmonary Disease, Chronic Obstructive"[Mesh]
	2	"Bronchitis, Chronic"[Mesh]
	3	"Pulmonary Emphysema"[Mesh]
	4	"Lung Diseases, Obstructive"[Mesh]
	5	obstruct* AND (pulmon* OR lung* OR airway* OR airflow* OR bronch* OR respirat*)
	6	COPD OR AECOPD OR COAD OR COBD OR AECB
	7	or/1-6
	8	"Respiration, Artificial"[Mesh]
	9	"Positive-Pressure Respiration"[Mesh]
	10	"Noninvasive Ventilation"[Mesh]
	11	"Ventilators, Mechanical"[Mesh]
	12	"Intubation, Intratracheal"[Mesh]
	13	(artificial OR mechanical OR non-invasive OR noninvasive) AND (ventilat* OR respirat*)
	14	or/8-13
	15	"Electric Stimulation Therapy"[Mesh]
	16	"Transcutaneous Electric Nerve Stimulation"[Mesh]
	17	"Electric Stimulation"[Mesh]
	18	(neuromuscular OR functional) AND electric*

	19	electrotherap* OR electromyostimulation OR electrostimulation OR (electric* AND stimulation)
	20	NMES OR FES OR TENS
	21	or/15-20
	22	and/7,14,21
CENTRAL (Cochrane Library)	1	MeSH descriptor: [Pulmonary Disease, Chronic Obstructive] explode all trees
	2	MeSH descriptor: [Bronchitis, Chronic] explode all trees
	3	MeSH descriptor: [Pulmonary Emphysema] explode all trees
	4	MeSH descriptor: [Lung Diseases, Obstructive] explode all trees
	5	obstruct* AND (pulmon* OR lung* OR airway* OR airflow* OR bronch* OR respirat*)
	6	COPD OR AECOPD OR COAD OR COBD OR AECB
	7	or/1-6
	8	MeSH descriptor: [Respiration, Artificial] explode all trees
	9	MeSH descriptor: [Positive-Pressure Respiration] explode all trees
	10	MeSH descriptor: [Noninvasive Ventilation] explode all trees
	11	MeSH descriptor: [Ventilators, Mechanical] explode all trees
	12	MeSH descriptor: [Intubation, Intratracheal] explode all trees
	13	(artificial OR mechanical OR non-invasive OR noninvasive) AND (ventilat* OR respirat*)
	14	or/8-13
	15	MeSH descriptor: [Electric Stimulation Therapy] explode all trees

	16	MeSH descriptor: [Transcutaneous Electric Nerve Stimulation] explode all trees
	17	MeSH descriptor: [Electric Stimulation] explode all trees
	18	(neuromuscular OR functional) AND electric*
	19	electrotherap* OR electromyostimulation OR electrostimulation OR (electric* AND stimulation)
	20	NMES OR FES OR TENS
	21	or/15-20
	22	and/7,14,21
EMBASE (Elsevier Databases)	1	"chronic obstructive lung disease"/exp
	2	"chronic bronchitis"/exp
	3	"lung emphysema"/exp
	4	"obstructive airway disease"/exp
	5	obstruct* AND (pulmon* OR lung* OR airway* OR airflow* OR bronch* OR respirat*)
	6	COPD OR AECOPD OR COAD OR COBD OR AECB
	7	or/1-6
	8	"artificial ventilation"/exp
	9	"positive pressure ventilation"/exp
	10	"noninvasive ventilation"/exp
	11	"mechanical ventilator"/exp
	12	"endotracheal intubation"/exp

	13	(artificial OR mechanical OR non-invasive OR noninvasive) AND (ventilat* OR respirat*)
	14	or/8-13
	15	"electrotherapy"/exp
	16	"transcutaneous electrical nerve stimulation"/exp
	17	"electrostimulation"/exp
	18	"functional electrical stimulation"/exp
	19	"neuromuscular electrical stimulation"/exp
	20	(neuromuscular OR functional) AND electric*
	21	electrotherap* OR electromyostimulation OR electrostimulation OR (electric* AND stimulation)
	22	NMES OR FES OR TENS
	23	or/15-22
	24	and/7,14,23
CINAHL Complete (EBSCOhost Research Databases)	1	MH "Pulmonary Disease, Chronic Obstructive+"
	2	MH "Bronchitis, Chronic"
	3	MH "Emphysema+"
	4	MH "Lung Diseases, Obstructive+"
	5	obstruct* AND (pulmon* OR lung* OR airway* OR airflow* OR bronch* OR respirat*)
	6	COPD OR AECOPD OR COAD OR COBD OR AECB
	7	or/1-6
	8	MH "Respiration, Artificial+"

	9	MH "Positive Pressure Ventilation+"
	10	MH "Ventilators, Mechanical"
	11	MH "Intubation, Intratracheal+"
	12	(artificial OR mechanical OR non-invasive OR noninvasive) AND (ventilat* OR respirat*)
	13	or/8-12
	14	MH "Electric Stimulation+"
	15	MH "Electrical Stimulation, Neuromuscular"
	16	MH "Transcutaneous Electric Nerve Stimulation"
	17	MH "Electrical Stimulation, Functional"
	18	(neuromuscular OR functional) AND electric*
	19	electrotherap* OR electromyostimulation OR electrostimulation OR (electric* AND stimulation)
	20	NMES OR FES OR TENS
	21	or/14-20
	22	and/7,13,21

Clinical trial registries

Clinicaltrials.gov

"Electric Stimulation Therapy" OR "Electric Stimulation" OR "Transcutaneous Electric Nerve Stimulation" OR "Neuromuscular Electrical Stimulation" OR "Functional Electrical Stimulation" | Interventional Studies | "Chronic Obstructive Pulmonary Disease" OR "Obstructive Lung Diseases"

ICTRP

("Chronic Obstructive Pulmonary Disease" OR "Obstructive Lung Diseases") AND ("Electric Stimulation Therapy" OR "Electric Stimulation" OR "Transcutaneous Electric Nerve Stimulation" OR "Neuromuscular Electrical Stimulation" OR "Functional Electrical Stimulation")

Grey literature

("Chronic Obstructive Pulmonary Disease" OR "Obstructive Lung Diseases") AND ("Electric Stimulation Therapy" OR "Electric Stimulation" OR "Transcutaneous Electric Nerve Stimulation" OR "Neuromuscular Electrical Stimulation" OR "Functional Electrical Stimulation")

Online resource 2. On-going studies

ID	Population	Interventions	Outcomes	Status
ChiCTR-IPR-16009845<br><br>* Could include COPD patients on ventilatory support	**Inclusion Criteria**: All included patients consistent with the diagnosis of 'global COPD prevention initiative (GOLD)' on acute exacerbation of COPD, aged 45 to 90 years old, and volunteer to participate in the study and signed the informed consent form.<br><br>**Exclusion Criteria**: Comorbidities that could limit exercise training, neuromuscular disease, pacemaker implantation, and being unable to understand the questionnaires and unable to cooperate.	**Intervention Group**: NMES on the quadriceps femoris muscle.<br><br>**Control Group**: sham NMES on the quadriceps femoris muscle.	- Muscle function<br>- Exercise capacity<br>- Cross sectional area of the quadriceps<br>- Length of hospital stay<br>- Antibiotic using time<br>- Dyspnoea<br>- D-dimer<br>- Complications<br>- IL-8<br>- TNF-a<br>- Lung function<br>- Arterial blood gas analysis	- Recruiting<br>- First posted: November 13, 2016<br>- Last updated posted: April 18, 2017

NCT03548870<br><br>* Unlikely to include COPD patients on ventilatory support	**Inclusion Criteria**: A diagnosis of COPD.<br><br>**Exclusion Criteria**: (1) Exercise contraindication; (2) Any musculoskeletal problems, cardiovascular or neurological comorbidities that limits exercise; (3) pH < 7,35; (4) Body temperature > 38°C; (5) cardiac frequency > 100 bpm at rest; (6) systolic blood pressure < 100 mmHg; (7) exacerbation during the study; (8) heart pacemaker or defibrillator; (9) Opiate treatment during the last 3 months.	**Intervention Group**: Patients will perform one Constant Work-Rate Exercise Test at 80% of maximum workload with low frequency TENS.<br><br>**Control Group**: Patients will perform one Constant Work-Rate Exercise Test at 80% of maximum workload with low frequency sham-TENS.	- Exercise tolerance<br>- Peripheral muscle oxygenation<br>- Dyspnoea<br>- Oxygen saturation<br>- Heart rate<br>- Muscular fatigue<br>- Oxygen consumption<br>- Carbon dioxide production<br>- Minute ventilation<br>- Tidal volume<br>- Respiratory rate<br>- Inspiratory capacity<br>- Respiratory quotient	- Not yet recruiting<br>- First posted: June 7, 2018<br>- Last updated posted: October 23, 2020

NCT03844711<br><br>* Could include COPD patients on ventilatory support	**Inclusion Criteria**: (1) Adults between 40 and 80 years old; (2) Smoking more than 10 pack-years; (3) Clinical diagnosis of COPD, GOLD 3 and 4; (4) Patients admitted for severe COPD exacerbation (appearance or worsening of dyspnea, appearance or worsening of cough and appearance or increase in sputum that may be purulent), two of which must be present to characterize exacerbation.<br><br>**Exclusion Criteria**: (1) Unstable ventricular arrhythmia; (2) Unstable angina; (3) Aortic stenosis; (3) Uncontrolled systemic arterial hypertension; (4) Epilepsy; (5) Undergoing hemodialysis; (6) Fever and / or infectious	**Intervention Group**: For transcutaneous electrical diaphragmatic stimulation, surface electrodes will be used that will be positioned at the transcutaneous motor points of the diaphragm.<br><br>**Control Group**: Ventilatory exercises, bronchial hygiene techniques, passive, active-assisted or active exercises for upper and	- Respiratory muscle strength (MIP; MEP)<br>- Lung function<br>- Diaphragm muscle mobility<br>- Electromyography of the diaphragm muscle<br>- Lower Limb Strength<br>- Body composition measure<br>- Dyspnoea<br>- Exercise capacity<br>- Systolic blood pressure<br>- Diastolic blood pressure<br>- Heart rate<br>- Respiratory rate	- Recruiting<br>- First posted: February 18, 2019<br>- Last updated posted: May 3, 2021

	disease; (7) Neoplasms; (8) Pacemaker; (9) At the time of the intervention:<br>Oxygen saturation below 90%; Obese patients; Refuse to participate in the survey.	lower limbs, and resistance exercises.	- Oxygen saturation<br>- Length of hospital stay	
NCT04704479<br><br>* Unlikely to include COPD patients on ventilatory support	**Inclusion Criteria**: (1) Men with stage II COPD Patients; (2) Aged from 55 to 65 years' old; (3) FEV_1/FVC less than 70% (Patients of moderate COPD (Stage II- GOLD criteria); (4) BMI 25.0-29.9 kg/m^2; (5) Tobacco smokers; (6) No history of infections or symptom exacerbations in the previous two months before the study; (7) Did not participate in any selective exercise program for the respiratory muscles before.<br><br>**Exclusion Criteria**: (1) Women; (2) Acute exacerbation that requires a change in	**Intervention Group**: Combined Russian and EMT.<br><br>**Control Group**: EMT only.	- MEP<br>- Lung function<br>- Dyspnoea<br>- Functional capacity<br>- COPD Assessment Test	- Recruiting<br>- First posted: January 11, 2021<br>- Last updated posted: January 11, 2021

pharmacological management or hospitalization; (3) An open injury affecting the application of surface electrodes of Russian current; (4) Asthmatic patient; (5) Implanted pacemaker; (6) Patients with chest infection; (7) Patients with pleural diseases; (8) Primary valvular disease; (9) History of spontaneous pneumothorax; (10) Clinically significant peripheral vascular disease; (11) Severe anemia; (12) BMI more than 29.9 kg/m2; (13) Previous lung surgery; (14) Long-term oxygen treatment; (15) Patients with chronic renal failure.; (16) Any cognitive impairment that interferes with prescribed exercise procedures; (17) Musculoskeletal or neurological limitation to physical exercise; (18) Any patient enrolled in an anther research study for at least 30 days.

BMI: Body mass index; **COPD**: Chronic obstructive pulmonary disease; **EMT:** Expiratory muscle training; **IL-8:** Interleukin-8; **FEV_1:** Forced expiratory volume in one second; **FVC**: Forced vital capacity; **MEP:** Maximal expiratory pressure; **MIP:** Maximal inspiratory pressure; **NMES:** Neuromuscular electric stimulation; **TENS:** Transcutaneous electrical nerve stimulation; **TNF-a:** Tumor necrosis factor alpha.

Online resource 3. Full text records not retrieved

Search resource	Title
Electronic databases	In the Press: Ventilation in the ICU
Electronic databases	Question 4-3. The role of non-invasive measurement of ventilation during exercise, electrostimulation of the quadriceps, and the work of breathing in the rehabilitation of patients with chronic obstructive pulmonary disease
Electronic databases	Question 4-3. Role of noninvasive ventilation associated with exercise, the electrostimulation of quadriceps, and eccentric muscle work in the rehabilitation of patients with COPD
Other metrhods	Effect of neuro-muscular electrical stimulation on muscle dysfunction in exacerbations of COPD

COPD: Chronic obstructive pulmonary disease; **ICU**: Intensive care unit.

Online resource 4A. Full-text records excluded from database and registry searches

Id	Title	Reference	Reason for exclusion
Abdellaoui 2011	Skeletal muscle effects of electrostimulation after COPD exacerbation: a pilot study	Eur Respir J 2011; 38: 781–788<br>* Identified from records of RCTs (Registration: NCT01167283 – Status: completed)	Population – unbundled outcome reporting for participants with COPD
Angelopoulos 2013	Acute microcirculatory effects of medium frequency versus high frequency NMES in critically ill patients - a pilot study	Ann Intensive Care 2013;3(1):39	Population - unbundled outcome reporting for participants with COPD
Azambuja 2019	NMES and transcutaneous electrical diaphragmatic stimulation in hospitalized patients with chronic cardiorespiratory diseases: a RCT	Journal of Respiratory and CardioVascular Physical Therapy 2019,7(2):3-12	Population - includes participants with COPD without ventilator support
Bonnevie 2017	Home-based NMES as an add-on to pulmonary rehabilitation does not provide further benefits in patients with COPD: a multicentre randomized trial	Arch Phys Med Rehabil 2018;99(8):1462-1470.<br>* Identified from records of RCTs (Registration: NCT02171377 – Status: completed)	Population - includes participants with COPD without ventilator support

Bonnevie 2019	Lumbar TENS to improve exercise performance in COPD patients	Eur Respir J 2019; 54: 1900784<br>* Identified from records of RCTs (Registration: NCT03312322 – Status: completed)	Study design - Participants are their own controls
Bouchla 2009	NMES as an alternative means of exercise for the critically ill	Archives of Hellenic Medicine 2009, 26(6):759-777	Study design - It is a narrative review
Cancelliero-Gaiad 2013	Acute effects of transcutaneous electrical diaphragmatic stimulation on respiratory pattern in COPD patients: cross-sectional and comparative clinical trial	Braz J Phys Ther 2013; 17(6): 547–555.<br>* Identified from records of RCTs (Registration: NCT01300442 – Status: completed)	Population - includes participants with COPD without ventilator support
Carkner 2022	NMES improves lower-limb strength after long-term bedrest: Case study in a mechanically-ventilated intensive care patient	Am J Respir Crit Care Med 183;2011:A4242	Study design - It is a case report
Castelain 2015	Early rehabilitation of COPD patients in ICU	No publication of results identified<br>* Identified from records of RCTs (Registration: NCT00628992 – Status: terminated)	Other - the study was terminated prematurely

Cooper 2015	An investigation into the effect of NMES and pulmonary rehabilitation in patients with COPD	No publication of results identified<br>* Identified from records of RCTs (Registration: ISRCTN46428072 – Status: completed)	Population - includes participants with COPD without ventilator support
Folz 2018	Abdominal functional electrical stimulation to reduce hyperinflation in COPD Patients	No publication of results identified<br>* Identified from records of RCTs (Registration: NCT02035228 – Status: completed)	Population - includes participants with COPD without ventilator support
Franssen 2019	Effects of a comprehensive, inpatient pulmonary rehabilitation programme in a cachectic patient with very severe COPD and chronic respiratory failure	Breathe (Sheff) 2019;15(3):227-233	Study design - It is a case report
Gerstenhaber 2012	Electrical stimulation of accessory muscles of respiration: Is there a role for enhancing weaning from mechanical ventilatory support in long term acute care?	Am J Respir Crit Care Med 2012;185:A3091	Study design - No control group

Greening 2017	Effects of neuromuscular stimulation on quadriceps in COPD	No publication of results identified<br>* Identified from records of RCTs (Registration: ISRCTN87439020 – Status: completed)	Population - includes participants with COPD without ventilator support
Gutiérrez-Arias 2021	Effect of NMES on the duration of mechanical ventilation	Respir Care 2021;66(4):679-685	Study design - It is a SR
Jones 2009	Effects of a 4-week Acu-TENS program on subjects with stable COPD	No publication of results identified<br>* Identified from records of RCTs (Registration: NCT00971490 – Status: completed)	Population - includes participants with COPD without ventilator support
Jones 2011	Acute effects of Acu-TENS on FEV_1 and blood β-endorphin level in COPD	Altern Ther Health Med 2011;17(5):8-13	Population - includes participants with COPD without ventilator support

Kho 2012	NMES for ICU-acquired weakness: protocol and methodological implications for a randomized, sham-controlled, phase II trial	Phys Ther 2012;92(12):1564-1579<br>* Identified from records of RCTs (Registration: NCT00709124 – Status: completed)	Population - the participants included do not have COPD
Kuang Cheng 2019	Electric muscle stimulation for patients with chronic respiratory failure	Results were reported on the RCTs registration webpage<br>* Identified from records of RCTs (Registration: NCT01930643 – Status: terminated)	Population - does not specify whether to include participants with COPD
Lau 2008	A single session of Acu-TENS increases FEV_1 and reduces dyspnoea in patients with COPD: a randomised, placebo-controlled trial	Aust J Physiother 2008;54(3):179-84	Population - includes participants with COPD without ventilator support
Lisy 2014	Overview of reviews: Mechanical interventions for the treatment and management of COPD	Int J Nurs Pract 2014;20(6):701-8	Study design - It is an overview of SR

Maddocks 2016	NMES to improve exercise capacity in patients with severe COPD: a randomised double-blind, placebo-controlled trial	Lancet Respir Med 2016; 4: 27–36<br>* Identified from records of RCTs (Registration: ISRCTN15985261 – Status: completed)	Population - includes participants with COPD without ventilator support
Medeiros 216	Physiotherapy intervention during level I of pulmonary rehabilitation on COPD: a SR	Open Respir Med J 2016;10:12-9	Study design - It is a SR
Medrinal 2018	Comparison of exercise intensity during four early rehabilitation techniques in sedated and ventilated patients in ICU: a randomised cross-over trial	Crit Care 2018;22(1):110	Population - unbundled outcome reporting for participants with COPD
Meesen 2007	The effect of NMES	No publication of results identified<br>* Identified from records of RCTs (Registration: NCT00442728 – Status: completed)	Outcome - does not report an outcome relevant to this SR
Meesen 2010	NMES as a possible means to prevent muscle tissue wasting in artificially ventilated and sedated patients in the ICU: A pilot study	Neuromodulation 2010;13(4):315-20; discussion 321	Population - unbundled outcome reporting for participants with COPD

Ngai 2009	Effect of Acu-TENS on airway obstructive disease	Thesis submitted in partial fulfilment of the requirements for the degree of Doctor of Philosophy	Population - includes participants with COPD without ventilator support
Ngai 2011	Acute effects of Acu-TENS on FEV_1 and blood B-endorphin level in COPD	Altern Ther Health Med 2011;17(5):8-13<br>* Identified from records of RCTs (Registration: NCT00922051 – Status: completed)	Population - includes participants with COPD without ventilator support
Öncü 2017	The effect of TENS in patients with acute exacerbation of COPD: RCT	J Clin Nurs 2017;26(13-14):1834-1844	Population - includes participants with COPD without ventilator support
Ozgultekin 2018	Acute systemic microcirculatory effects of high frequency NMES in critically ill patients	Intensive Care Medicine Experimental 2018, 6(Suppl 2):40	Outcome - does not report an outcome relevant to this SR

Routsi 2009	Electrical muscle stimulation prevents critical illness polyneuromyopathy in ICU patients - A randomized parallel intervention trial	Intensive Care Med 2009, 35, S133	Population - the participants included do not have COPD
Routsi 2010	Electrical muscle stimulation prevents critical illness polyneuromyopathy: a randomized parallel intervention trial	Crit Care 2010;14(2):R74<br>* Identified from records of RCTs (Registration: NCT00882830 – Status: completed)	Population - the participants included do not have COPD
Routsi 2010b	Electrical muscle stimulation prevents critical illness polyneuromyopathy: a randomized parallel intervention trial	Crit Care 2010;14(2):R74	Population - the participants included do not have COPD
Sillen 2013	Effects of resistance training or NMES in patients with COPD	Respir Med 2013;107(8):1186-1194<br>* Identified from records of RCTs (Registration: NTR2322 – Status: completed)	Population - includes participants with COPD without ventilator support
Vieira 2014	NMES improves clinical and physiological function in COPD patients	Respir Med 2014;108(4):609-20	Population - includes participants with COPD

			without ventilator support
Vivodtzev 2012	Functional and muscular effects of NMES in patients with severe COPD: a RCT	Chest 2012;141(3):716-725	Population - includes participants with COPD without ventilator support
Yen-Huey Chen 2017	Effects of electrical stimulation on hospitalization outcomes in patients with prolong mechanical ventilator	Am J Respir Crit Care Med 2017, 195<br>* Conference proceedings	Population - unbundled outcome reporting for participants with COPD
Yen-Huey Chen 2019	NMES in patients with prolonged mechanical ventilation	Respir Care 2019;64(3):262-271<br>* Identified from records of RCTs (Registration: NCT02227810 – Status: completed)	Population - unbundled outcome reporting for participants with COPD
Yen-Huey Chen 2020	Effects of muscle electrical stimulation on respiratory muscle in patients with prolonged mechanical ventilation	Eur Respir J 2020, 56<br>* Conference proceedings	Population - unbundled outcome reporting for participants with COPD

Yen-Huey Chen 2021	NMES in patients with prolonged mechanical ventilation	No publication of results identified<br>* Identified from records of RCTs (Registration: NCT04076475 – Status: completed)	Population - does not specify whether to include participants with COPD
Yun Han 2010	Effect of Chinese medicine intestine adjusting therapy on patients with respiratory failure caused by acute exacerbation of COPD and undergoing noninvasive ventilation	Zhongguo Zhong Xi Yi Jie He Za Zhi 2010;30(8):814-8	Intervention - electroacupuncture as an NMES modality
Zanotti 2011	NMES in COPD	No publication of results identified<br>* Identified from records of RCTs (Registration: NCT00677690 – Status: completed)	Population - includes participants with COPD without ventilator support
Zhiling Zhao 2020	Inspiratory plus expiratory NMES versus diaphragm pacing for rehabilitation in severe COPD patients: a RCT	DOI: 10.21203/rs.3.rs-58117/v2 Preprint published at www.researchsquare.com<br>* Identified from records of RCTs (Registration: ChiCTR2000032681 – Status: completed)	Population - includes participants with COPD without ventilator support

COPD: Chronic obstructive pulmonary disease; **ICU:** Intensive care unit; **NMES:** Neuromuscular electrical stimulation; **RCT:** Randomized controlled trial; **SR:** Systematic review; **TENS:** Transcutaneous electrical nerve stimulation.

Online resource 4B. Full-text records excluded from the search by other methods

Id	Title	Reference	Reason for exclusion
Abdellaoui 2011	Skeletal muscle effects of electrostimulation after COPD exacerbation: a pilot study	Eur Respir J 2011; 38: 781–788	Population – unbundled outcome reporting for participants with COPD
Abu-Khaber 2013	Effect of NMES on prevention of ICU acquired muscle weakness and facilitating weaning from mechanical ventilation	Alexandria Journal of Medicine 2013;49(4):309-315	Population - the participants included do not have COPD
Chaplin 2013	Neuromuscular stimulation of quadriceps in patients hospitalised during an exacerbation of COPD: A comparison of low (35 Hz) and High (50 Hz) frequencies	Physiother Res Int 2013;18(3):148-56	Population - includes participants with COPD without ventilator support
Chen 2018	Effects of early passive motion and NMES on ICU acquired weakness in mechanically ventilated patients	Chinese Journal of Rehabilitation Medicine 2018;33(2):146-150	Population – unbundled outcome reporting for participants with COPD

Bustamante 2010	Muscle training with repetitive magnetic stimulation of the quadriceps in severe COPD patients	Respir Med 2010;104(2):237-45	Population - includes participants with COPD without ventilator support
Bustamante 2013	Neuromuscular magnetic stimulation of the quadriceps muscle after COPD exacerbations	Eur Respir J 2013 42: P3572	Population - includes participants with COPD without ventilator support
Dall Acqua 2012	NMES with Russian current for expiratory muscle training in patients with COPD	J Phys Ther Sci 2012;24:955–959,	Population - includes participants with COPD without ventilator support
Dall Acqua 2017	Use of NMES to preserve the thickness of abdominal and chest muscles of critically ill patients: A randomized clinical trial	J Rehabil Med 2017;49(1):40-48	Population - the participants included do not have COPD

Dos Santos 2020	NMES combined with exercise decreases duration of mechanical ventilation in ICU patients: A RCT	Physiother Theory Pract 2020;36(5):580-588	Population - the participants included do not have COPD
Giavedoni 2012	NMES prevents muscle function deterioration in exacerbated COPD: A pilot study	Respir Med 2012;106(10):1429-34	Population - includes participants with COPD without ventilator support
Gigliotti 2004	Effect of NMES as an adjunct to an exercise training program in patients with severe COPD	Eur Respir J 2004; 24: Suppl. 48, 1681	Population - includes participants with COPD without ventilator support
Kho 2012	NMES for ICU-acquired weakness: protocol and methodological implications for a randomized, sham-controlled, phase II trial	Phys Ther 2012;92(12):1564-1579<br>* Identified from records of RCTs (Registration: NCT00709124 – Status: completed)	Population - the participants included do not have COPD

Koutsioumpa 2018	Effect of TENS on myopathy in intensive care patients	Am J Crit Care 2018;27(6):495-503	Population – unbundled outcome reporting for participants with COPD
Kucio 2016	Evaluation of the effects of NMES of the lower limbs combined with pulmonary rehabilitation on exercise tolerance in patients with COPD	J Hum Kinet 2016; 54: 75–82	Population - includes participants with COPD without ventilator support
Lopez 2017	Results between two electrostimulation modalities in acute exacerbations of COPD patients	Eur Respir J 2017 50: PA2562	Population - includes participants with COPD without ventilator support
McCaughey 2019	Abdominal functional electrical stimulation to assist ventilator weaning in critical illness: a double-blinded, randomised, sham-controlled pilot study	Crit Care 2019;23(1):261	Population - the participants included do not have COPD

Malaguti 2009	Constant or adjustable intensity of electrical current does not elicit different level of quadriceps femoris fatigue during NMES sessions in patients with COPD	Am J Respir Crit Care Med 2009;179:A3402	Population - includes participants with COPD without ventilator support
Martín-Salvador 2016	Physical therapy intervention during hospitalization in patients with acute exacerbation of COPP and pneumonia: A RCT	Med Clin (Barc) 2016;146(7):301-4	Population - includes participants with COPD without ventilator support
Meesen 2010	NMES as a possible means to prevent muscle tissue wasting in artificially ventilated and sedated patients in the ICU: A pilot study	Neuromodulation 2010;13(4):315-20; discussion 321	Population - unbundled outcome reporting for participants with COPD
Neves 2014	NMES improves clinical and physiological function in COPD patients	Eur Respir J 2014 44: P1289	Population - unbundled outcome reporting for participants with COPD
Öncü 2017	The effect of TENS in patients with acute exacerbation of COPD: RCT	J Clin Nurs 2017;26(13-14):1834-1844	Population - includes participants with COPD

			without ventilator support
Routsi 2010	Electrical muscle stimulation prevents critical illness polyneuromyopathy: a randomized parallel intervention trial	Crit Care 2010;14(2):R74	Population - the participants included do not have COPD
Sonia 2012	Effect of Acu-TENS on pulmonary functions in patients with acute exacerbation of COPD	Indian J Physiother Occup Ther 2012;6(4):115-119	Population - includes participants with COPD without ventilator support
Tasdemir 2015	NMES as an adjunct to endurance and resistance training during pulmonary rehabilitation in stable COPD	Expert Rev Respir Med 2015;9(4):493-502	Population - includes participants with COPD without ventilator support
Vieira 2014	NMES improves clinical and physiological function in COPD patients	Respir Med 2014;108(4):609-20	Population - includes participants with COPD

			without ventilator support
Yen-Huey Chen 2019	NMES in patients with prolonged mechanical ventilation	Respir Care 2019;64(3):262-271	Population - unbundled outcome reporting for participants with COPD
Yun Han 2010	Effect of Chinese medicine intestine adjusting therapy on patients with respiratory failure caused by acute exacerbation of COPD and undergoing noninvasive ventilation	Zhongguo Zhong Xi Yi Jie He Za Zhi 2010;30(8):814-8	Intervention - electroacupuncture as an NMES modality

COPD: Chronic obstructive pulmonary disease; **ICU:** Intensive care unit; **NMES:** Neuromuscular electrical stimulation; **RCT:** Randomized controlled trial; **SR:** Systematic review; **TENS:** Transcutaneous electrical nerve stimulation.
